# MicroRNA-4316 inhibits gastric cancer proliferation and migration via directly targeting VEGF-A

**DOI:** 10.1186/s12935-020-1132-3

**Published:** 2020-02-22

**Authors:** Haithm Mousa, Menglang Yuan, Xinsheng Zhang, Xiaomeng Li, Abdullah Shopit, Marwan Almoiliqy, Mohammed Alshwmi, Aisha Al-Dherasi, Yue Xu, Yunfei Zuo

**Affiliations:** 1 Department of Clinical Biochemistry, College of Laboratory Diagnostic Medicine, Dalian Medical University, Dalian, 116044 China; 2 Department of General Surgery, The Second Affiliated Hospital of Dalian Medical University, Zhongshan Road No. 467, Shahekou District, Dalian, 116023 Liaoning China; 3 Pharmacology Department, Dalian Medical University, Dalian, 116044 China; 4 Clinical Laboratory, The First Hospital of Dalian Medical University, Dalian, 116011 China; 5 Center of Genome and Personalized Medicine, Institute of Cancer Stem Cell, Dalian Medical University, Dalian, 116011 China

**Keywords:** Gastric cancer (GC), miR-4316, VEGF-A, Proliferation, Metastasis

## Abstract

**Background and aims:**

microRNAs (miRNAs) have been reported to regulate proliferation and migration by down-regulating the expression of target genes. The aims of this study were to investigate whether miR-4316 inhibited proliferation and migration by downregulating vascular endothelial growth factor A (VEGF-A) and its clinical significance in gastric cancer (GC).

**Methods:**

The clinical tissues of the GC patients for miR-4316 and VEGF-A were detected by qRT-PCR. The protein levels of VEGF-A and c-Met were determined by western blotting. Cell Proliferation, migration, and colony forming assays were conducted to show whether miR-4316 affects proliferation by CCK-8, migration by transwell, wound healing and colony formation assays. The bioinformatic methods and luciferase reporter assay were applied to detect the relationship between miRNA and VEGF-A on its targeting 3-untranslated regions (3-UTRs). CCK-8, colony formation, wound healing, and transwell assay were performed to explore the function of miR-4316.

**Results:**

The results of qRT-PCR indicated that miR-4316 expression level was significantly downregulated in human GC tissues and GC cell lines compared with their control. miR-4316 inhibited proliferation, migration and colony formation in GC cell lines by reducing VEGF-A. And western blot results indicated that miR-4316 significantly inhibited GC through repressing VEGF-A and c-Met. The investigation of Luciferase assay indicated that VEGF-A is a direct target gene of miR-4316.

**Conclusions:**

miR-4316 suppressed proliferation and migration of GC through the VEGF-A gene. MiR-4316 acts as a tumor suppressor by targeting VEGF-A and this indicated that MiR-4316 might be a potential therapeutic target for GC.

## Background

Gastric cancer is still one of the most common and deadly tumors in the world. Nevertheless, the incidence rate has steadily declined [1]. Gastric cancer is also one of the major cancers that are most affected by behavior and might be preventable [2]. Previously, it was reported that about 90% of cancer deaths are caused by developed primary metastatic tumors such as gastric adenocarcinoma [3]. Recently, In one large cancer statistics study, about 26,240 cases and 10,800 deaths of gastric cancer were estimated in 2018 [4]. Thus, The widespread tumor migration and invasion contribute to a high rate of death. There are numerous stimulatory and inhibitory factors which play a role important in cancer angiogenesis for growth and metastasis of gastric cancers [5]. Despite the progress in the treatment of GC, the effects of conventional therapies for GC, including chemotherapy and radiation, on prolonged survival are limited. Therefore, we need to revealing novel molecules to understand the mechanism of the metastasis. In recent years, along with studies of the tumor cell signal pathways, targeted medicine has become a promising strategy for the treatment of GC. Hence, studies on the novel pathways of GC tumorigenesis provide potential candidates to improve the prognosis of GC. Because of low survival rates and decrease treatment options, particularly in developing countries, lead to reducing incidence might be the key to reducing mortality [6]. Additionally, Gastric cancers and other cancers have been reported with risk factors involving smoking, *H. pylori* infection and male gender [7]. Angiogenesis is crucial for tumor growth and progression because contains a lot of network of blood vessels supplying the tumor’s oxygen and nutrients and discard the metabolic waste from the tumor. Therefore, the angiogenesis process plays a critical role in the growth and metastasis of the tumors [8].

MIRNA is endogenous, small single-strand noncoding RNA that has an ability to promote or suppress the expression of many mRNAs genes. It involved in cell signaling pathways essential for malignant occurrence and progression, such as cell proliferation, motility, apoptosis, and angiogenesis [9, 10]. It has been reported that miRNAs regulate more than 60% of the proteins translation. Many biological processes were regulated by miRNAs and play an important role in modulating phenotype, cell migration, differentiation, and cell cycle [11–13]. Moreover, Emerging evidence suggests that abnormal expression of miRNA is closely related to cancer development through regulating oncogene and tumor suppressors [14]. Therefore, miRNAs might have great potential as prognostic indicators and therapeutic targets in many types of malignancies [15]. Initially, It was known that both of miR-221 and miR-222 regulate the angiogenic properties of human umbilical vein endothelial cells, overexpression of miR-221 and miR-222 effect p27(Kip1), p57(kip2) and c-kit expression and inhibit endothelial cell-promoted tube formation [16]. Recently, it has been indicated that miR-4316 acts as a tumor suppressor or oncogene in two different cancers [17–20].

VEGF-A is the most potent pro-angiogenic factor that acts directly on endothelial cells to induce endothelial cell proliferation, migration, survival, and ultimately angiogenesis that promotes tumor growth. VEGF-A expression can be mediated through the PI3K/Akt/HIF-1α signaling pathway. It has been reported that miR-15a, miR-16, and miR-503 inhibit tumor angiogenesis by targeting VEGF-A [21–23]. PIK3C2α belongs to class II PI3Ks and can recruit Akt by phosphorylating PIP2, which plays an important role in angiogenesis. PIK3C2α was also found to be essential for S1P1-induced S1P1 internalization, endosomal Rac1 activation, and cell migration in endothelial cells [24, 25]. However, it is unclear whether the PIK3C2α/Akt/VEGF-A signaling pathway is regulated by miRNA.

In this study, we identified a novel tumor-suppressive miRNA, miR-4316, and investigated its functional role in gastric cancer compared with non-gastric cancer. Moreover, we identified VEGF-A as a direct target for miR-4316. Our results not only revealed that overexpressed VEGF-A in gastric cancer cell lines could be the result of down-regulation of miR-4316 but also suggested an important role for the loss of miR-4316 in the angiogenesis and migration of gastric cancer cells and its potential for a future therapeutic application.

## Methods and materials

### Human tissues, cell lines, and cell culture

A total of 12 pairs of gastric cancer tissues of patients and their matched adjacents normal tissues were obtained from patients under underwent surgical resections at the second hospital affiliated to Dalian Medical University. Use of patient samples was approved by the ethics committee of our Institute. GES-1 is a normal gastric epithelium cell line (GES-1, SGC-7901, BGC-823 and MGC-803) were purchased from the Institute of Biochemistry and Cell Biology of the Chinese Academy of Sciences (Beijing, China). The gastric cancer cell lines (SGC-7901, BGC-823 and MGC-803) were cultured in RPMI1640 and GES-1 was cultured in Dulbecco’s modified Eagle’s medium DMEM (Hyclone) both of media were supplemented with 10% FBS, penicillin (100 U/ml) and streptomycin (100 μg/ml), in a CO_2_ incubator (Forma Scientific).

### Construction and transfection

The siRNA oligos for VEGF-A were constructed according to the previous report. Target sequences were aligned to the human genome database in a BLAST search to ensure that the choosing sequences were not highly homologous with other genes. And miR-4316-mimic and miR-4316-inhibitor with Negative control were constructed by (Genepharma, China). SGC-7901 and BGC-823 cells were plated in six-well plates and cultured in drug-free medium. At 60% confluence, both cells were washed twice with PBS, grew in 2 ml of RPMI1640 without antibiotics. Using Lipofectamine™ 2000 reagent and 100 pmol of siRNA plasmids; VEGF-A sense: 5′-UCCGCAGACGUGUAAAUGUTT3′; VEGF-A-antisense: 5′-ACAUUUACACGUCUGCGGATT-3′; N.C-VEGF-Asense: 5′-UUCUCCGAACGUGUCACGUTT-3′; anti-sense: 5′ ACGUGACACGUUCGGAGAATT-3′, miR-4316 (mimic, inhibitor and NC), miRBase Accession number named as MIMAT0016867.

miR-4316-mimic-sense: 5′-GGUGAGGCUAGCUGGUG-3′; anti-sense: 5′-CCAGCUAGCCUCACCUUU-3′; miR-4316-inhibitor, sense: 5′ CACCAGCCUAGCCUCACC-3′; N.C: sense: 5′CAGUACUUUUGUGAGUACAA-3′, were transfected into the cells according to the manufacturer’s instructions. The cells transfected with vector alone were served as a negative control. Forty-eight hours later, cells were plated in RPMI1640 growth medium for 48 h. The expression levels of VEGF-A and miR-4316 were analyzed by western blot analysis and qRT-PCR respectively for further experiments.

### RNA extraction and (qRT-PCR)

Cells were transfected with (si-VEGF-A, miR-4316-mimic and miR-4316-inhibitor), or their negative control. After 48 h, the total RNA was extracted using TRIzol (RNAiso Plus, Takara, China) Reagent according to the manufacturer’s instruction. Reverse transcription reactions were performed by using mi-RNA qRT-PCR Detection Kit Briefly, the extracted RNA was reverse-transcribed in the presence of a poly-A polymerase with an oligo-dT adaptor. QRT-PCR was then carried out using SYBR Green Mix. The cells and total RNA in each transfection group were extracted using RNA reagent kit (Invitrogen Inc., Carlsbad, CA, USA). The primers of miR-4316, VEGF-A and GAPDH were designed and synthesized by company. The reactions were as follows, VEGF-A-forward: 5′-GCAGAAGGAGGGCAGAATC-3′; VEGF-A-reverse: 5′-ACACTCCAGGCCCTCGTCATT-3′; GAPDH-forward: 5′-GTCAAGGCTGAGAACGGGAA-3′; GAPDH-reverse: 5′-AAATGAGCCCCAGCCTTCTC-3′; miR-4316: 5′-GGTGGGCTGCTGGG-3′; U6: 5′-CTCGCTTCGGCAGCACA-3′. The total RNA was reversely converted into cDNA through the specific stem-loop reverse transcription primer by using miRcute plus miRNA First-strand cDNA Synthesis Kit (TIANGEN, China). The reverse transcription system was conducted in 10 μl, and the reaction conditions were as follows: 42 °C for 60 min, 95 °C for 3 min and 85 °C for 10 min. The miRcute-SYBR MicroRNA Assays was applied in the qRT-PCR, and the reaction conditions were as follows: 42 cycles of pre-denaturation for 2 min at 94 °C, denaturation for 20 s at 94 °C, annealing for 34 s at 60 °C, and extension for 30 s at 72 °C. However, after extracted total RNA, for mRNA, cDNA synthesis was transcribed using kit according to the manufacturer’s (Tiangen, Takara, China). The reaction system included 0.6 μl PCR upstream primer, 0.6 μl PCR downstream primer, 2 μl cDNA template, 6.8 μl SYBR Premix and 10 μl RNase free H_2_O. The relative expressions were calculated according to 2^−ΔΔCt^, normalized U6 and GAPDH as the internal control, with Hsa-miR-4316 and VEGF-A as the target gene.

### Western blotting

The cells (SGC-7901 and BGC-823) in each transfected group were cracked after transfected 48 h (miR-4316-mimic, miR-4316-inhibitor and N.C) or (si-VEGF-A, miR-4316-mimic + si-VEGF-A, miR-4316-inhibitor + si-VEGF-A, and N.C) the total protein was extracted from the cells and lysed by using kits (KeyGen, Nanjing, China). Followed assessing of total protein concentration with BCA assay protein kit (Beyotime Biotechnology, Shanghai, China) according to the manufacturer’s instruction. After lysates of equal cell protein concentration were mixed with loading buffer, sodium dodecyl sulfate-polyacrylamide gel electrophoresis (SDS-PAGE) was performed to isolate it. Proteins that separated by electrophoresis were transferred to a nitrocellulose filter membrane 0.22 μm, and the membranes were blocked in non-fat dry 5% powdered milk mixture of Tris-buffered saline (TBS-T-20) and Polysorbate 20 (also known as Tween-20) solution was added for 1 h at room temperature. The membranes were incubated for overnight at 4 °C with monoclonal anti-rabbit VEGF-A primary antibodies (dilution 1:500; BOSTER BIOTECH), monoclonal anti-mouse c-Met primary antibodies (dilution 1:200; Santa Cruz Biotechnology) and anti-mouse polyclonal β-actin primary antibodies (dilution 1:1000; Elabscience). The filter membrane was washed with the TBS-T-20 buffer 3 times, then followed with incubated with goat-anti-rabbit (dilution 1:800) and goat-anti-mouse (dilution 1:2000) labeled with a secondary antibody for 1 h at room temperature. Then the secondary antibodies were washed by TBS-T-20-Tween six times each time 5 min. The membranes were exposed by chemiluminescence instrument according to the manufacturer’s instructions and followed exposure to ImageQuant LAS 500 (GE Healthcare Life Sciences, Shanghai, China) for 2 to 12 min.

### Cell count cell-8 (CCK-8)

Cell proliferation was monitored by ELISA with CCK-8 kit (dojindo chemical technology, Shanghai company), according to the manufacturer’s instruction. Briefly, 100 μl of cell suspension from each subgroup at density (3 × 10^3^ cells/well) were seeded in a 96-well plates after transfected the cells (SGC-7901 and BGC-823) for 24 h with (miR-4316-mimic, miR-4316-inhibitor and N.C) or (si-VEGF-A, miR-4316-mimic + si-VEGF-A,  miR-4316-inhibitor + si-VEGF-A, and N.C) then incubated for 24 h, 48 h, and 72 h. Subsequently, 10 μl of CCK-8 kit solution was added to per well and incubated for 3 h. After adding a kit solution for 3 h, the absorbance was measured by (BIO-RAD) instrument at 450 nm using a Microplate spectrophotometer (xMark, BIO-RAD, CA, USA). The experiments were repeated three times.

### Wound healing assay

A wound-healing assay was performed to assess cell migration. Briefly, 2 × 10^5^ transfected cells by (miR-4316-mimic, miR-4316-inhibitor and N.C,) or (si-VEGF-A, miR-4316-mimic + si-VEGF-A, miR-4316-inhibitor + si-VEGF-A, and N.C) were seeded in a six-well plate and cultured in RPMI1640 for 24 h. A linear wound was created by scraping the confluent cell mono-layer by 10 μl tips size. Cells were washed twice with PBS and cultured in RPMI1640 serum-free for additional 24 h. Wound closure was measured by a microscope (Olympus, Japan) photographing five randomly selected fields at the time of wounding at 0 and 48 h after wounding.

### Transwell assays

For the transwell migration assay, the Gastric cancer cells lines (40 × 10^4^ of SGC-7901 and 20 × 10^4^ of BGC-823) were trypsinized and seeded into the upper chamber of each insert (8 mm pore size; Corning, Cambridge, USA), containing 200 μl of medium free fetal bovine serum after 48 h transfection (miR-4316-mimic, miR-4316-inhibitor and N.C) or (si-VEGF-A, miR-4316-mimic + si-VEGF-A, miR-4316-inhibitor + si-VEGF-A and N.C). Lower chambers were supplemented with 1% fetal bovine serum (600 μl). The non-migrated cells in the membrane of the upper surface were removed with a cotton tip after 24 h incubation at 37 °C, while the cells on the lower surface which migrated through the pores were stained with 0.1% crystal violet (Biosharp, Hefei, China) for 30 min. The numbers of migrated cells were observed from digital images captured on an Olympus microscope (Olympus Inc.) and calculated using ImageJ software.

### Colony formation assay

Five-hundred cells were seeded into six-well plates after transfected the cells BGC823 and SGC7901 by (miR-4316-mimic, miR-4316-inhibitor, and N.C) or (si-VEGFA, miR-4316-mimic + si-VEGF-A, miR-4316-inhibitor + si-VEGF-A, and N.C). After incubation for 2 weeks, colonies were fixed by ethanol for 15 min, followed by staining with crystal violet for 20 min then formative colonies were counted.

### Immunofluorescence stain assay

SGC7901 and BGC823 cell lines were transfected by (miR-4316-mimic, miR-4316-inhibitor and N.C) or (si-VEGF-A, miR-4316-mimic + si-VEGF-A, miR-4316-inhibitor + si-VEGF-A and N.C) for 48 h, the media were removed then followed with 3 washes of 1× PBS. The cells fixed by using paraformaldehyde 4% at room temperature for 15 min. Subsequently, washed by 1× PBS to removed residual para-formaldehyde. Then, permeabilized cells with 0.1% Triton X-100 made in PBS solution for 15 min at room temperature. Then washed three times by 1× PBS 5 min per time. Block solution BSA 5% incubated at 37 °C for 1 h. Incubated in 37 °C for 1 h in primary antibody anti-rabbit diluted in 1× PBS v/v (1:50), then incubated in 4 °C for overnight. And Alexa-Fluor 594-conjugated Goat Anti-Rabbit IgG (H + L) (protein-tech-USA) was added after washed primary antibody three times 5 min per time stored in the dark for 1 h, washed by 1× PBS 3 times one time 5 min. Finally added DAPI (Sigma) for 7 min then washed three times 5 min per time and observed under the fluorescence microscope (Olympus Inc.).

### Luciferase reporter assay

To confirm the regulation of VEGF-A by miR-4316. The dual-luciferase reporter plasmid fused with the wild-type or mutant 3′-UTR segment of human VEGF-A was obtained from Gene-Pharma (Shanghai, China). SGC7901 and BGC823 cells were both seeded into 24-well plates for 24 h before transfection. miR-4316-mimic or N.C, and the target gene 3′-UTR reporter gene plasmid were cotransfected into the cells with, 20 nM miR-4316-mimic or N.C and 0.1 ng of the VEGF-A Wild-type or Mutant 3′-UTR reporter plasmids with Lipofectamine 2000 reagent (Invitrogen, Carlsbad, CA, USA). After 48 h, the cells were harvested with passive lysis buffer and the luciferase activity was calculated by the Dual-Luciferase Reporter Assay System (Promega, Madison, WI, USA) according to the manufacturer’s instructions. The Rinella luciferase signals were normalized to firefly luciferase signal for every individual analysis.

### Statistical analysis

All statistical Data analyses were performed to analyze in vitro and human samples by using Graph Pad Prism 7.0 software. The statistical significant was analyzed by t-test to comparisons between the groups. The data are shown as the mean ± SE; P < 0.05 was considered to indicate a significant difference.

## Results

### The expression of miR-4316 is downregulated in clinical specimens and GC cell lines

**Fig. 1:**
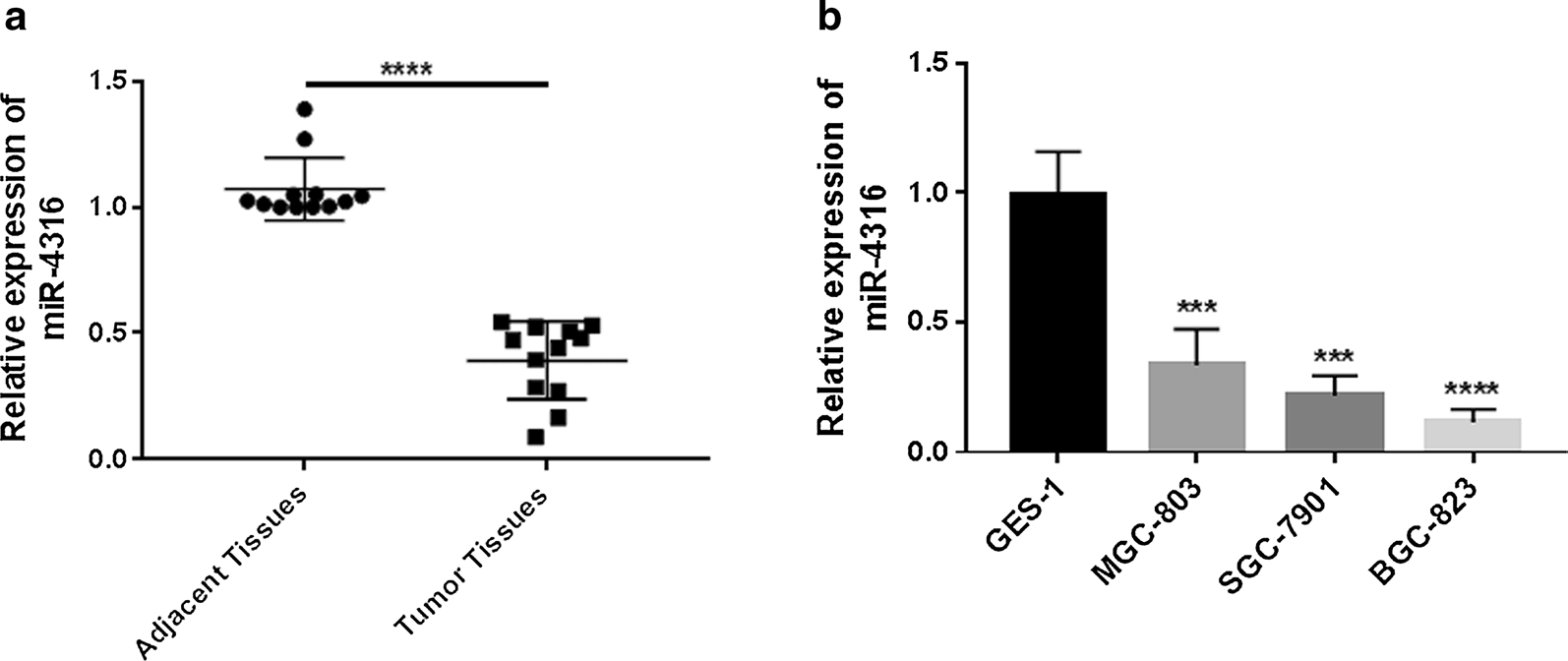
The expression of miR-4316 in clinical specimens and GC cell lines. **a** qRT-PCR analysis showed that miR-4316 expression was significantly downregulated in gastric cancer tissues compared with adjacent non-tumor tissues n = (12). **b** Lower level of miR-4316 was observed in GC cell lines, including GES-1as normal cell line as well as SGC-7901, MGC-803 and BGC-823 as GC cell lines. ***P < 0.001 and ****P < 0.0001

The expression level of miR-4316 in gastric cancer tissues and their adjacent normal tissues were determined by qRT-PCR. Our results suggested that the expression level of miR-4316 was significantly downregulated in gastric cancer tissues than their adjacent normal tissues. Thus, the expression level of miR-4316 in three human gastric cell lines SGC-7901, MGC-803 and BGC-823 were significantly downregulated compared with normal epithelial cell line GES-1as shown in Fig. 1a, b.

### The role of miR-4316 in inhibition of gastric cancer cells proliferation, migration and colony formation

**Fig. 2:**
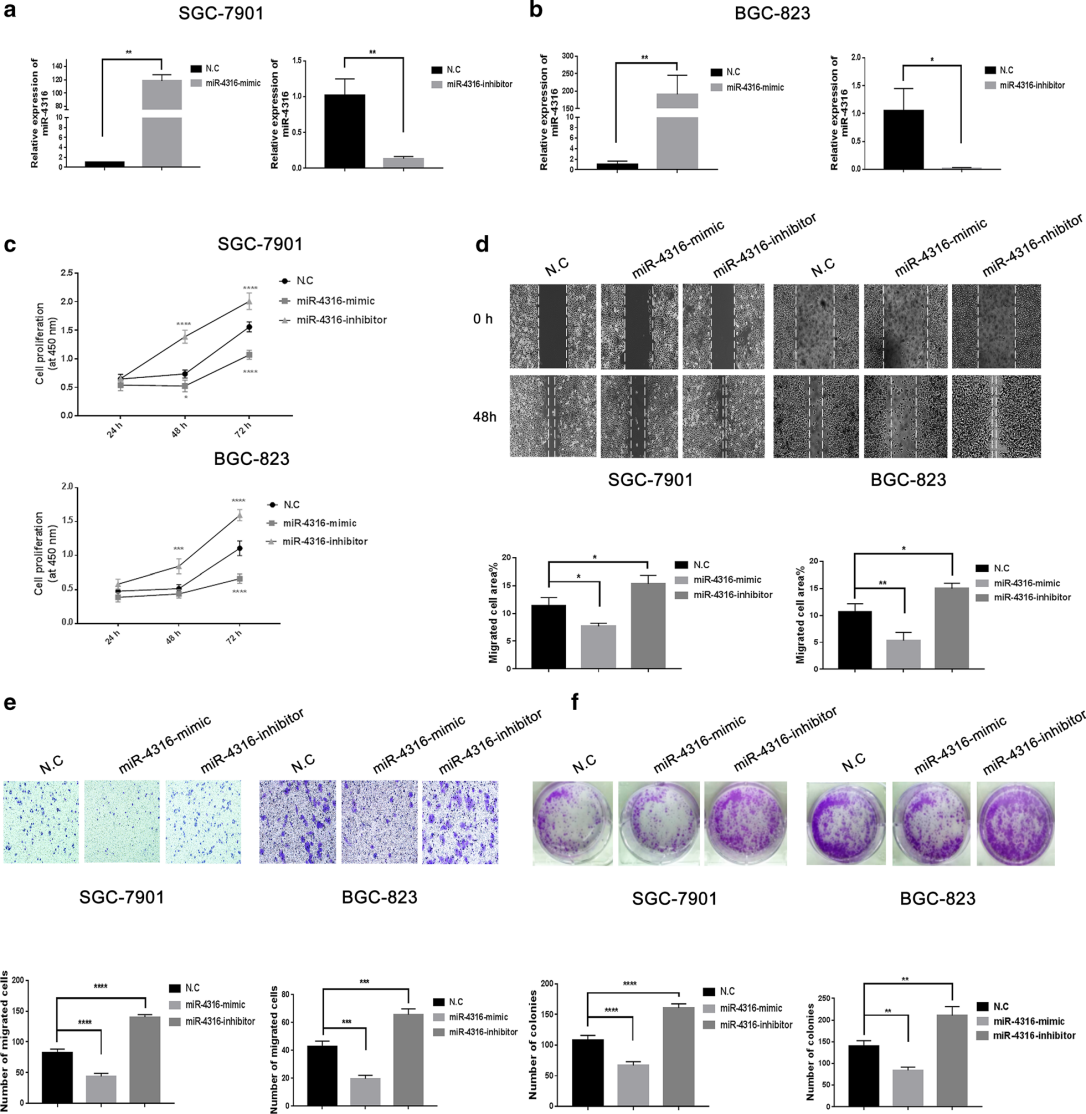
Over-expression of miR-4316 inhibited of gastric cells proliferation, migration and colony formation. **a**, **b** MiR-4316 levels were increased and decreased in SGC-7901 and BGC-823 cells transfected with miR-4316-mimic and miR-4316-inhibitor. **c** SGC-7901 and BGC-823 cells were transfected with miR-4316-mimic, miR-inhibitor, or N.C for 0, 24, 48, and 72 h. The cell proliferation was performed by CCK-8. **d** SGC-7901 and BGC-823 cells were transfected with miR-4316-mimic, miR-inhibitor, or N.C for 48 h. Then migrated area was shown in **d**. **e** SGC-7901 and BGC-823 cells were transfected with miR-4316-mimic, miR-4316-inhibitor, or N.C for 24 h. The cells migration was detected by transwell assay. The migrated cells were accounted per field ×20 for SGC-7901 and ×40 for BGC-823 as shown in **e**. **f** Colony formation assay of the changes in proliferation capacity of SGC-7901 and BGC-823 cells after transfection with miR-4316-mimic, miR-inhibitor, or N.C for 14 days. *P < 0.05, **P < 0.01, ***P < 0.001 and ****P < 0.0001

The efficiency of the transfection was investigated using qRT-PCR. After 48 h transfection with miR-4316-mimic, miR-4316-inhibitor or N.C, the expression of miR-4316 in SGC-7901 and BGC-823 was higher using its mimic. Whereas, the expression of miR-4316 was decreased in (SGC-7901 and BGC-823) using its miR-inhibitor. This confirmed that miR-4316-mimic and miR-4316-inhibitor can effectively regulate the expression in gastric cancer cells as shown respectively (Fig. 2a, b).

CCK-8 assay results indicated that the proliferation of (SGC-7901 and BGC-823) cell lines were significantly suppressed after transfected with of miR-4316-mimic as shown in Fig. 2c; furthermore, the results of wound healing showed wound healing distances were bigger than N.C as shown in Fig. 2d. Furthermore, the effect of miR-4316 was examined by transwell, our results demonstrated that miR-4316-mimic markedly decreased numbers of migrated cells as shown in Fig. 2e and in addition, colony formation was significantly weakened as shown in Fig. 2f. These results suggested that miR-4316 acts as an onco-suppressive miRNA in GC progression.

To more clarify the role and function of miR-4316 in GC cell lines we evaluated the biological properties by transfected the cells SGC-7901 and BGC-823 with miR-4316-inhibitor and N.C. The results showed that miR-4316-inhibitor was markedly promoting proliferation, migration and colony formation in GC cell lines as shown in Fig. 2c–f. Briefly, these findings suggested that inhibition of miR-4316 in GC cell lines promotes proliferation, migration and colony formation.

### VEGF-A is a novel target gene of miR-4316 and suppressed progression of GC cells through regulating VEGF-A

**Fig. 3:**
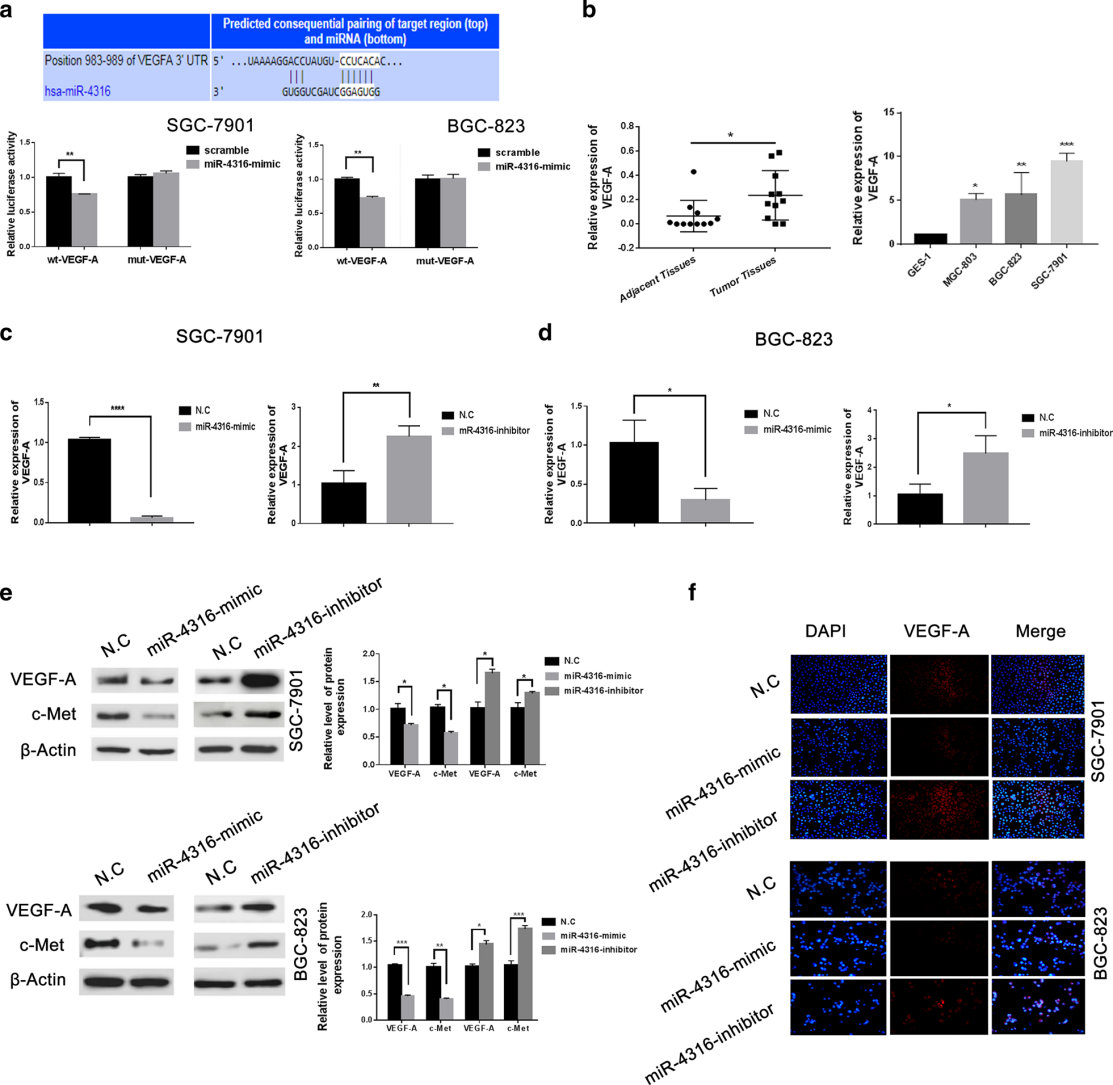
VEGF-A is a novel target gene of miR-4316 and miR-4316 inhibits VEGF-A gene expression via targeting VEGF-A 3′-UTR. **a** Luciferase reporter assays demonstrated that miR-4316-mimic reduced WT-VEGF-A activity in SGC-7901 and BGC-823 cells compared to MUT-VEGF-A. **b** qRT-PCR used to detect the expression of VEGF-A in 12 gastric cancer tissues and their adjacent normal tissues and Relative expression of VEGF-A in SGC-7901 and BGC-823 gastric cancer cell lines was determined by qRT-PCR compared with normal cells GES-1. **c**, **d** The expression of VEGF-A in GC cell lines SGC-7901 and BGC-823 cells was determined by qRT-PCR after transfection with miR-4316-mimic, inhibitor, or N.C. **e** The protein level of VEGF-A and c-Met were detected after 48 h transfection by miR-4316-mimic, miR-4316-inhibitor, or N.C in SGC-7901 and BGC-823 cells by western blot. **f** After transfection with miR-4316-mimic, miR-4316-inhibitor, or N.C in SGC-7901 and BGC-823 cells for 48 h, the fluorescence signal of VEGF-A was observed by fluorescence microscopy. Merged images were overlapped for nuclear staining by DAPI was blue color and for VEGF-A was red color. *P < 0.05, **P < 0.01, ***P < 0.001 and ****P < 0.001

To find the underlying mechanism of miR-4316 in gastric cancer, we investigated potential targets of miR-4316 using three databases prediction softwares (TargetScan7.2, mirDIP, and miRWalk) to validate that VEGF-A is a direct target of miR-4316 (Fig. 3a). The target gene of miR-4316 was verified by luciferase reporter gene assay. First, miR-4316 regulation of VEGF-A transcription was demonstrated in SGC-7901 and BGC-823 cells. Then, to further verify the regulatory effect of miR-4316 on VEGF-A, SGC-7901 and BGC-823 cells were chosen and the results shown in Fig. 3a, this result suggested that VEGF-A gene is a target of miR-4316. The expression level of VEGF-A was significantly upregulated in gastric cancer tissues than adjacent normal tissues. In addition, at the cellular level, the obtained results were similar. Whereas, the expression level of VEGF-A was significantly upregulated in gastric cancer cell lines SGC-7901, MGC-823 and BGC-823 compared with normal gastric epithelial cell lines GES-1 (Fig. 3b). The two cell lines (SGC-7901 and BGC-823) have used during this study because of both of two cell lines with low expression of miR-4316.

Additionally, qPCR results showed that VEGF-A expression was significantly decreased in miR-4316-mimic transfected cells when compared with the NC group, while it was highly expressed in miR-4316-inhibitor as shown in Fig. 3c, d. Furthermore, we performed western blot to examine the VEGF-A level and c-Met protein level, the results showed that miR-4316-mimic significantly reduced VEGF-A level and c-Met protein level, In contrast, miR-4316-inhibitor significantly elevated VEGF-A level and c-Met protein level as showed in Fig. 3e. By, immunofluorescence assay the results revealed that VEGF-A expression was markedly low after transfection with miR-4316-mimic in SGC-7901 and BGC-823 cells. Moreover, in contrast, miR-4316-inhibitor lead to enhanced the level of VEGF-A expression in the cells as shown in Fig. 3f.

### The role of miR-4316 and VEGF-A in GC cells

#### MiR-4316-reduces VEGF-A suppressing proliferation and cell growth in GC cells

**Fig. 4:**
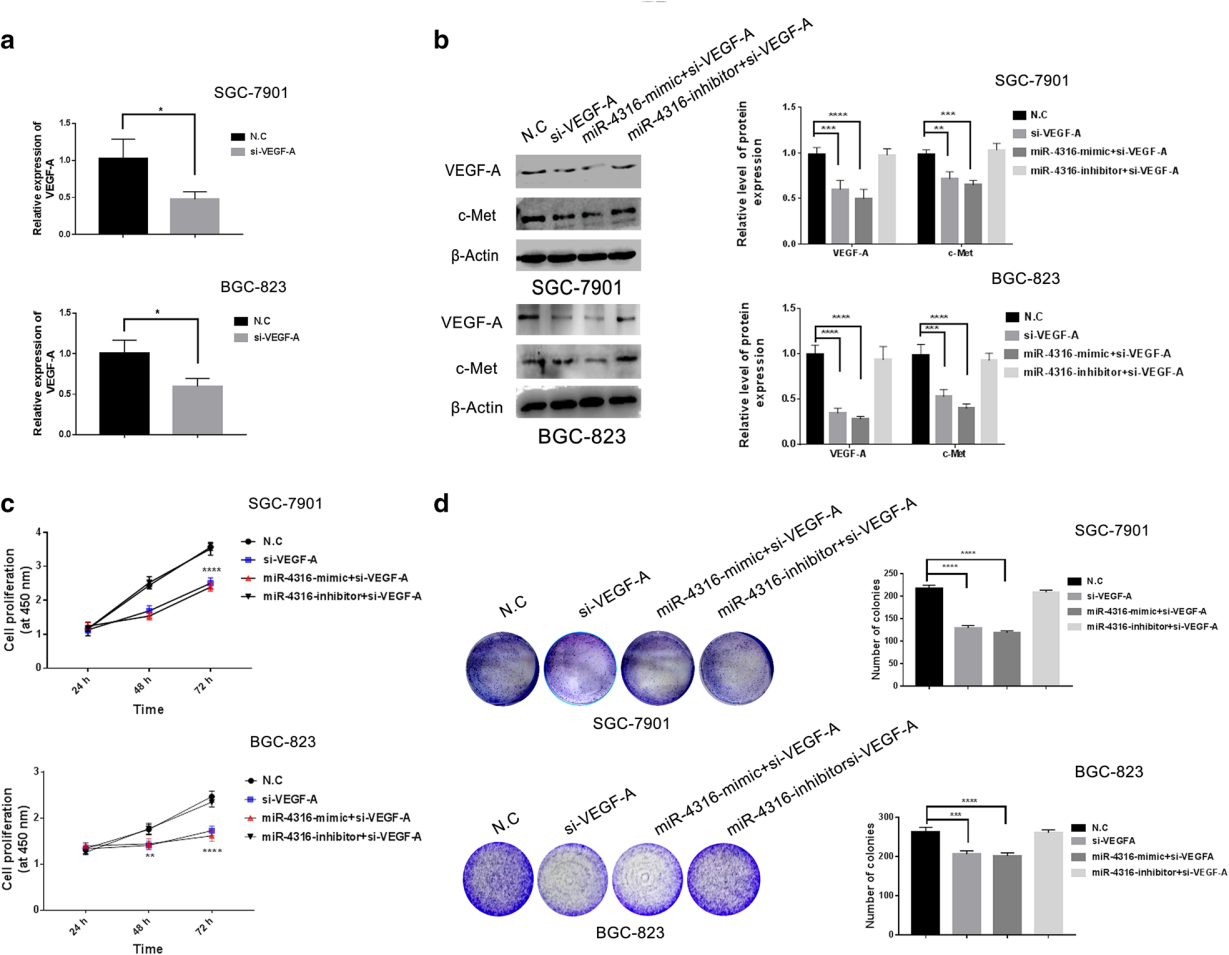
MiR-4316-reduced VEGF-A and suppressed proliferation of GC cells. **a** qRT-PCR confirmed the VEGF-A expression after knocked down with si-VEGF-A or N.C in SGC-7901 and BGC-823. **b** By Western blot, the quantitative level of VEGF-A and c-Met protein expression was decreased after transfection and co-transfection in SGC-7901 and BGC-823 with si-VEGF-A, miR-4316-mimic + si-VEGF-A or miR-4316-inhibitor plus si-VEGF-A or N.C. **c**, **d** The proliferation in SGC-7901 and BGC-823 was detected by CCK-8 and colony formation assay after transfection and co-transfection with si-VEGF-A, miR-4316-mimic plus si-VEGF-A, miR-4316-inhibitor plus si-VEGF-A or N.C. *P < 0.05, **P < 0.001, ***P < 0.001 and ****P < 0.0001

To further verify the effect of the transfection with si-VEGF-A we used qRT- PCR to detect the efficiency of si-VEGF-A. Then our results showed that VEGF-A expression was markedly reduced compared with N.C as showed in Fig. 4a. To more explore the functional relationship between miR-4316 and VEGF-A, we transfected and co-transfected SGC-7901 and BGC-823 cells with si-VEGF-A, miR-4316-mimic + si-VEGF-A, and miR-4316-inhibitor + si-VEGF-A, the results showed that the protein expression of VEGF-A and c-Met was significantly decreased in the cells transfected with si-VEGF-A, and co-transfected with miR-4316-mimic + si-VEGF-A groups compared with N.C groups. On contrary, the protein expression of VEGF-A and c-Met was no significance in co-transfected with miR-4316-inhibitor + si-VEGF-A compared with N.C groups, confirmed by Western blot as described in Fig. 4b. To more confirmation, the CCK-8 results showed that the cell proliferation in si-VEGF-A, miR-4316-mimic + si-VEGF-A groups was significantly reduced in both of SGC-7901 and BGC-823 cell lines compared with N.C groups for 48 and 72 h. Whereas, the cell proliferation in cells co-transfected with miR-4316-inhibitor + si-VEGF-A was no significance compared with N.C groups as shown in Fig. 4c. To further clarify, the results of colony formation assay showed that the numbers of colonies were significantly decreased in transfected and cotransfected SGC-7901 and BGC-823 cells with si-VEGF-A, and miR-4316-mimic + si-VEGF-A compared with N.C after 14 days and were no significance in co-transfected with miR-4316-inhibitor + si-VEGF-A compared with N.C as showed (Fig. 4d).

#### miR-4316 inhibits VEGF-A reducing GC progression and migration

**Fig. 5:**
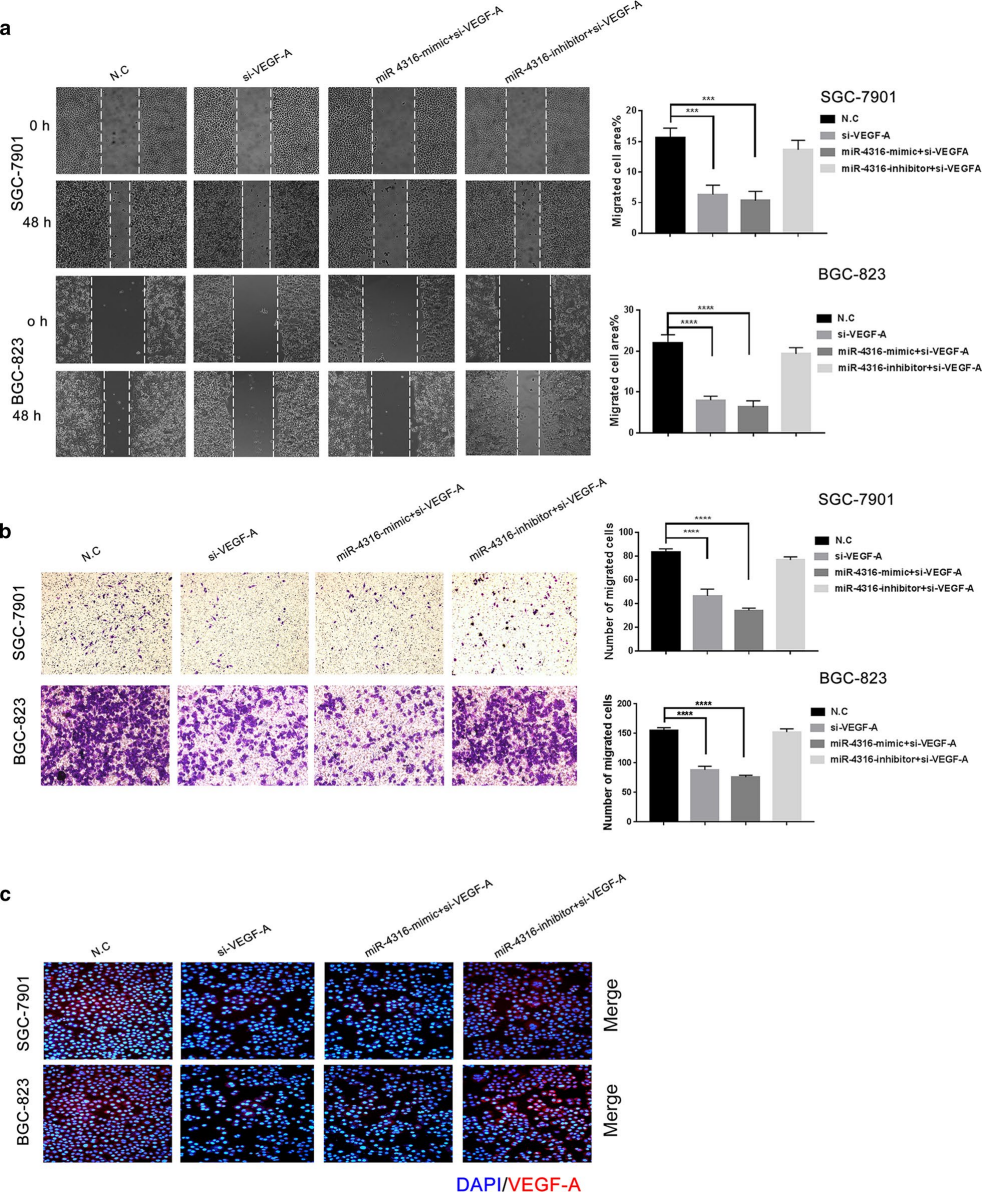
miR-4316 inhibits VEGF-A reducing GC progression and migration. **a** SGC-7901 and BGC-823 cells were transfected with with si-VEGF-A, miR-4316-mimic + si-VEGF-A or miR-4316-inhibitor plus si-VEGF-A or N.C for 48 h. Then migrated area was measured in **a**. **b** SGC-7901 and BGC-823 cells were transfected with si-VEGF-A, miR-4316-mimic + si-VEGF-A or miR-4316-inhibitor + si-VEGF-A or N.C for for 24 h. The cells migration was detected by transwell assay. The migrated cells were accounted per field ×20 for SGC-7901 and ×40 for BGC-823 as shown in **b**. **c** After transfection with si-VEGF-A, miR-4316-mimic + si-VEGF-A or miR-4316-inhibitor + si-VEGF-A or N.C for 48 h, the fluorescence signals of VEGF-A were observed by fluorescence microscopy. Merged images were overlapped for nuclear staining by DAPI was blue color and for VEGF-A was red color. ***P < 0.001 and ****P < 0.0001

**Fig. 6:**
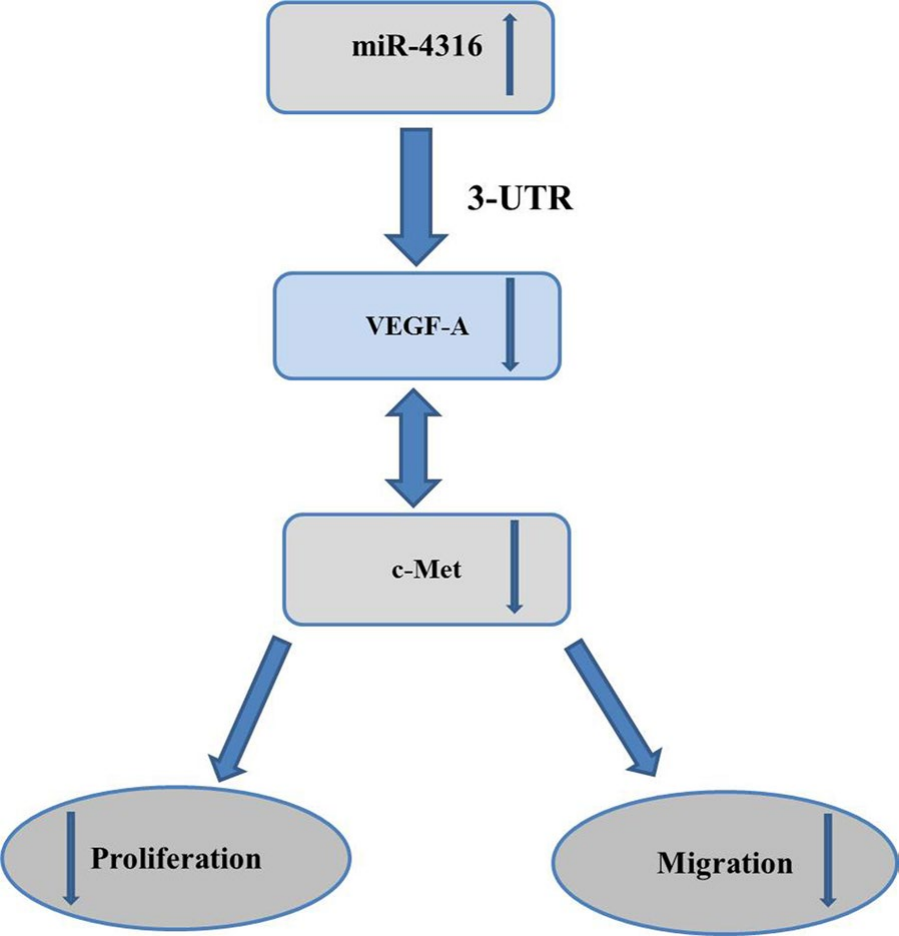
A schematic representation of the molecular mechanism of miR-4316 inhibits GC proliferation and migration through VEGF-A And c-Met (the relation between VEGF-A And c-Met have demonstrated previously)

Furthermore, the results revealed that transfection of si-VEGF-A alone, and co-transfection of both miR-4316-mimic + si-VEGF-A, indicated that the migration and transwell were significantly decreased compared with N.C. And co-transfection of both miR-4316-inhibitor + si-VEGF-A faild to decrease the wound healing and transwell compared with N.C as shown in Fig. 5a, b. Therefore, these findings indicated that VEGF-A is a functional target of miR-4316. In immunofluorescence assay VEGF-A protein expression was lower in transfected si-VEGF-A SGC-7901 and BGC-823 cell lines, and co-transfection of both miR-4316-mimic and si-VEGF-A SGC-7901 and BGC-823 cell lines compared with N.C. Whereas, co-transfection of both miR-4316-inhibitor and si-VEGF-A could not changed the VEGF-A protein expression compared with N.C as shown in Fig. 5c. In addition, a brief diagram that outlines the above-described regulatory interaction is shown in Fig. 6.

## Discussion

The main function of miRNA’s in cancer is via regulation of the gene at post-transcription as tumor cell development, metastatic, differentiate, and angiogenetic processes [26]. MIRNA acts as either oncogene or tumor suppressor could participate in regulating the expression of the genes related to migration and angiogenesis. In this study, we found that miR-4316 was low expressed in GC tissues and gastric cell lines. Additionally, miR-4316 upregulation was able to suppress gastric cancer cells proliferation and migration in vitro. MIR-4316 was reported to inhibit tumor progression in breast cancer, papillary thyroid cancer, bladder cancer, and hepatocellular carcinoma [17–20]. On the contrary, in early colon cancer, miR-4316 and miR-506 expression were elevated in peripheral blood of early colon cancer [27]. Other studies, were reported on others MiRNA’s which participate in cancer diseases. For instance, microRNA-140-5p inhibits invasion and angiogenesis of breast cancer via targeting VEGF-A [28]. Additionally, miR-101 suppresses angiogenesis and metastasis of nasopharyngeal carcinoma cell through directly inhibiting ITGA3 [29], and miR-203 abolished cervical cancer angiogenesis and growth of the tumor by targeting VEGF-A [30]. Therefore, miR-4316 might role in various types of tumor. In this study, we described the biological significance and the effects of miR-4316 dysregulating on proliferation and migration in human gastric cancer cell lines. Our findings are consistent with previous studies which reported in breast cancer, hepatocellular carcinoma bladder cancer and papillary thyroid glands [17–20]. However, the role of miR-4316 in GC needs further investigation. Here, we found that miR-4316 interacts with VEGF-A in gastric cell lines SGC-7901 and BGC-823. MiRNA modulates its target gene by binding on its complementary binding sites of mRNA 3′-UTR, and lead to inhibiting translating or degradation. In this study, the luciferase reporter gene assay verified that VEGF-A was a target gene of miR-4316.

Furthermore, we detected VEGF-A as a novel direct target gene of miR-4316 in gastric cancer cell lines. The VEGF gene is present on chromosome p21.3 and contains at least 8 exons and 7 introns. It has been found that VEGF-A expression was elevated in lymph node metastasis, liver metastasis, vascular distribution and poor prognosis in stomach cancer [31–33]. It has been reported that VEGF-A was enhanced proliferation, migration and cell division in GC [34]. The most potential of the pro-angiogenic factor is VEGF. Clinical observation has confirmed that VEGF-A is often over-expressed in a variety of tumors, leading to increased poor prognosis and neovascularization [35]. A meta-analysis explored that VEGF-A expression is an unfavorable prognostic factor in NSCLC [36]. In recent years, bevacizumab VEGF-A antagonists have been safely used in human alone or with chemotherapy combined [37]. Another previous study was found the interaction between VEGF-A and c-Met signaling in human Schwann cells and vestibular schwannomas [38], which consistent our study. Therefore, we hypothesized that VEGF-A is one of the miR-4316 target genes. According to the results of a luciferase reporter assay, qRT-PCR and western blot analysis, in our present study, VEGF-A was identified as a target gene of miR-4316. Meanwhile, our results demonstrated that the knockdown of VEGF-A inhibits c-Met and suppressed SGC-7901 and BGC-823 cell growth, migration, and colony formation in vitro. Based on these findings, we recommend a comprehensive analysis The expression status of miR-4316 and VEGF-A may improve our accuracy in identifying patients at high risk of poor prognosis, thus providing useful information for clinical management. MiR-4316 expression can be used as a prognostic index to predict the survival and recurrence of GC patients.

## Conclusions

In summary, the present study demonstrated that miR-4316 can significantly suppress GC cells proliferation and migration by directly targeting and down-regulating VEGF-A. These findings indicate that miR-4316 could serve as a tumor gene in GC. The newly identified miR-4316/VEGF-A help to further clarify this molecular mechanism which regulates metastasis and progression in gastric cancer and represents a novel strategy for prognosis and therapy against gastric cancer.

## Data Availability

All data generated or analyzed during this study are included in this article.

## Abbreviations

GC: Gastric cancer
miR-4316: MicroRNA-4316
VEGF-A: Vascular endothelial growth factor- A
qRT-PCR: Quantitative reverse transcription PCR
